# A Practical Multifaceted Approach to Selecting Differentially Expressed Genes

**Published:** 2008-01-15

**Authors:** Yingye Zheng, Margaret Pepe

**Affiliations:** 1,2 Fred Hutchinson Cancer Research Center, 1100 Fairview Avenue N., M2-B232 Seattle, WA 98109; 2 Department of Biostatistics, University of Washington, Box 357232 Seattle, WA 98195

## Abstract

Consider a gene expression array study comparing two groups of subjects where the goal is to explore a large number of genes in order to select for further investigation a subset that appear to be differently expressed. There has been much statistical research into the development of formal methods for designating genes as differentially expressed. These procedures control error rates such as the false detection rate or family wise error rate. We contend however that other statistical considerations are also relevant to the task of gene selection. These include the *extent* of differential expression and the *strength of evidence* for differential expression at a gene. Using real and simulated data we first demonstrate that a proper exploratory analysis should evaluate these aspects as well as decision rules that control error rates. We propose a new measure called the *mp*-value that quantifies strength of evidence for differential expression. The *mp*-values are calculated with a resampling based algorithm taking into account the multiplicity and dependence encountered in microarray data. In contrast to traditional *p*-values our *mp*-values do not depend on specification of a decision rule for their definition. They are simply descriptive in nature. We contrast the *mp*-values with multiple testing *p*-values in the context of data from a breast cancer prognosis study and from a simulation model.

## Introduction

In a gene expression array experiment, the expression levels of thousands of genes are monitored simultaneously. In cancer research the purpose of such a study is often to identify transcripts that show differential expression levels in cancer tissues as compared to normal tissues. This information may help to pinpoint the biological processes for cancer or to discover cDNAs encoding proteins that could be useful for cancer screening or diagnosis. Another common purpose is to compare gene expression in subjects with good and poor prognosis after being diagnosed with cancer. This information may help predict outcome for cancer patients and develop more specific treatment strategies. For example, in a study concerning gene expression profiling and clinical outcome of breast cancer (Van’t Veer, 2002), tumor tissue from 34 patients who developed distant metastases within 5 years and 44 patients who were free of disease for at least 5 years were analyzed to compare the hybridizations on an array of 25,000 cDNAs.

Statistical analysis of data from such studies is challenging. Moreover, it is important to recognize that an appropriate statistical approach depends on the scientific objectives of the study. In this article, we consider microarray studies that are aimed to explore a large pool of genes and select for more careful investigation a subset of genes that are differentially expressed. There has been much research into formal multiple hypothesis testing procedures for designating genes as differentially expressed. Procedures that control error rates have been a main focus of statistical research. Dudoit, Shaffer and Boldrick (2002) provide a review. Less attention however has been paid to other statistical aspects of the analysis that we feel are important. For example, the form and extent of differential expression at a gene are often important. If the difference in gene expression is not of sufficient magnitude or form to be useful, then such a gene should not be selected. Another issue is to characterize the strength of evidence for differential expression at a gene. In this paper we discuss the many aspects of assessing differential gene expression. We also propose a new descriptive measure, a probability that quantifies the strength of evidence for differential expression in a natural and intuitive fashion. In data analysis section we illustrate with real and simulated data how extent and evidence for differential expression can be used in conjunction with formal multiple hypothesis testing procedures to select genes for further study.

## Selecting Genes

### Error controlling decision rules

Recognizing the exploratory nature of gene finding studies and the high potential for erroneous conclusions based on standard univariate hypothesis testing procedures, statisticians have spent much effort on developing multivariate hypothesis testing procedures that control error rates. The multiple hypothesis testing paradigm is usually formulated as follows. Each of the *m* genes on the microarray is considered to correspond to a single hypothesis test. Rejecting an hypothesis test is equivalent to claiming that the gene is differentially expressed. In Table 1 we suppose *m*_0_ of the *m* genes are not differentially expressed, i.e. are null hypotheses. Denote by *R* the number of rejected hypotheses, V_0_ the number of false positives, and *V*_1_ the number of false negatives. Only *m* and *R* are observable quantities.

An appropriate test procedure aims to keep both *V*_0_ (the type I error) and *V*_1_(the type II error) small. In the univariate setting, the usual strategy is to pre-specify an acceptable type I error rate, α, then seek a decision rule with the smallest type II error among those with type I error α. To generalize to the multivariate setting, the approach is to define a multiple testing procedure in terms of an adjusted *p*-value, *p̃_j_*, for hypothesis *j*. The adjusted *p*-value, *p̃_j_*, is different from the individually unadjusted *p*-value *p_j_* in that *p̃_j_* takes all other hypothesis tests that are involved into consideration. One then rejects *H_j_* if *p̃_j_* ≤ α.

The adjusted *p*-values are derived so that some type I error rate is controlled at level α. The family-wise error rate (*FWER*), is defined as

(1) $$FWER=P(V_{0}\geq 1),$$

is the probability of at least one false positive (type I error). The step-down algorithm of Westfall and Young (1993) is an example of a multiple testing procedure that controls *FWER*. The procedure defines the jth adjusted *p*-value as *p̃_j_* = *P*[min_1≤_*_l_*_≤_*_m_* *P_l_* ≤ *p_j_|* $H_{0}^{c}$]. Here $H_{0}^{c}$ denotes the complete null hypothesis, where all the null hypotheses are true (i.e. *m* = *m*_0_) and *P_l_* is the unadjusted *p*-value for the *l*^th^ hypothesis denoted with capital letter here because it is a random variable. The joint distribution of ( *P*_1_, ..., *P*_m_) can be estimated by permuting the columns of the gene by array data matrix. This algorithm thus takes into account the potential dependence structure amongst genes but requires the so-called ‘subset pivotality’ property that is described in Dudoit, Shaffer and Boldrick (2002) (a complex and unintuitive notion). The popular but extremely conservative Bonferroni procedure defines the adjusted *p*-values as *p̃_j_* = *p_j_*/*m*. The Westfall and Young approach is less conservative. Recently, even less stringent procedures have been proposed (Dudoit et al. 2004; van der Laan et al. 2004; Lehmann and Romano, 2003) that are designed to control the generalized family-wise error rate (*gFWER*):

(2) $$gFWER=P(V_{0}\geq c).$$

Benjamini and Hochberg (1995) suggested a multiple testing procedure that aims to control a different type I error rate, the false discovery rate (*FDR*). In their definition,

(3) $$FDR=E(V_{0}/R|R>0)P(R>0)$$

The concept of *FDR* is appealing in the context of gene discovery. It is the expected proportion of false positives among genes for which *H*_0_ is rejected, an intuitive and directly useful quantity. Moreover it can be less stringent than controlling *FWER* or *gFWER*.

Several procedures have been proposed to control *FDR*. For example, Benjamini and Hochberg (1995) described a linear step-up procedure. Suppose we order the unadjusted *p*-values as *p*_(1)_ ≤ *p*_(2)_ ... ≤ *p*_(*m*)_, with corresponding ordered null hypotheses *H*_(1)_, *H*_(2)_, ..., *H*_(*m*)_. The adjusted *p*-value for *H*_(_*_j_*_)_ is ${\tilde{p}}_{\left(j\right)}^{BH}$ = min*_k=j,...m_* {min( $\frac{m}{k}$*P*_(*k*)_,1}. We reject *H*_(1)_, ..., *H*_(_*_k_*_)_ for *k* = max {*j*: ${\tilde{p}}_{\left(j\right)}^{BH}$ ≤ α} for a desired *FDR* level α. It can be shown that under some assumptions, the procedure yields *FDR* = α * *m*_0_/*m*, which is ≤ α (Benjamini and Yekutieli, 2001). When *m*_0_/*m* is substantially smaller than 1, it is tempting to consider an adaptive procedure so that *FDR* is controlled exactly at level α. Storey (2002) suggested to first estimate *m*_0_, and reject *H*_(1)_, ..., *H*_(*k*)_ for *k* = max{ *j*: ${\tilde{p}}_{\left(j\right)}^{BH}$ *m̂_0_/*m* ≤ α }. To estimate *m*_0_, Storey suggested

(4) $${\hat{m}}_{0}(\lambda )=\frac{\sum_{i=1}^{m}I\{p_{I}\geq \lambda \}}{1-\lambda }$$

where λ is in the interval (0, 1) and can be chosen using cross-validation, for example. The adaptive procedure is usually more powerful because it is less conservative, being based on the bound $\frac{m}{{\hat{m}}_{0}}$ α rather than α for ${\tilde{p}}_{\left(j\right)}^{BH}$.

Other formal error rate controlling procedures have been proposed (see Dudoit, Shaffer and Bolderick (2004) for examples) but the Westfall and Young (1993), Benjamini and Hochberg (1995) and Storey (2002) procedures mentioned above are currently most popular.

### Extent of differential expression

The fundamental component of any hypothesis testing procedure is the test statistic. Suppose that for gene *g,* expression data { $Y_{gj}^{C}$, *j* =1, ... *n_C_*} are measured on *n_C_* normal tissues and { $Y_{gj}^{D}$*,i* = 1, ... *n_D_*} cancer tissues. The test statistic implicitly defines the metric by which differential expression is quantified. Genes are typically ranked from highest (rank = 1) to lowest (rank = *m*) according to the test statistic. Thus, the whole meaning of differential expression between the populations of cancer subjects and non-cancer subjects is based on the test statistic. The larger the statistic is, the more differentially expressed {*Y_gi_,i* = 1, ..., *n_D_*} versus {*Y_gj_, j* = 1, ... *n_C_*} are considered. Genes that rank high with one measure of differential expression (test statistic) may rank low when another test statistic is used to quantify differential expression.

Despite its crucial role in selecting genes, there has been little discussion about what constitutes an appropriate test statistic. The Welch’s *t*-statistic is most commonly applied

$$W=({\bar{Y}}_{D}-{\bar{Y}}_{C})/\sqrt{\frac{S_{D}^{2}}{n_{D}}+\frac{S_{C}^{2}}{n_{C}}}$$

where *Ȳ* and *s*^2^ denote sample means and variances. We suspect that widespread familiarity with the *t*-test among biologists is primarily responsible for its popularity. As far as we know it has not been promoted as more appropriate than other two-sample statistics for the purposes of quantifying differential expression.

On the other hand there have been some arguments put forth for alternatives to the *t*-test (Lyons-Weiler et al. 2004; Pepe et al. 2003). For example, Lyons-Weiler et al. (2004) argued that while the *t*-test had been widely used for identifying population-level biomarkers, it could miss markers that might be important to a subset of patients. They developed a permutation percentile separability (PPST) test to identify important genes that are dysregulated in only a fraction of patients. For simplicity, suppose that larger values of *Y_g_* are associated with cancer. One might consider the sensitivity and specificity of classification to cancer based on the expression level: ‘*Y_g_* > threshold.’ The receiver operating characteristic (*ROC*) curve, a plot of the sensitivity versus 1-specificity with all possible thresholds, can be used to characterize the separation between the distributions of the gene expression levels for cancerous tissues and for normal tissues. Many summary measures of discrimination that are commonly used in *ROC* curve analysis can be considered. The “tail rank statistic” is the true positive rate (*TPR* or sensitivity) of the classifier that uses as threshold, the 100 × (1 – *f*_0_) percentile of *Y_g_* in the non-cancer population, denoted by $Z_{g}^{C}(f_{0})$. If values below $Z_{g}^{C}(f_{0})$ are considered within normal range, the *TPR* is the proportion of cases with abnormal expression at gene *g*. By definition the false positive rate (*FPR* = 1–specificity) of this rule is *f*_0_. We write the statistic as

$$TPR_{g}(f_{0})=\sum_{i=1}^{n_{D}}I[Y_{gi}^{D}\geq {\hat{Z}}_{g}^{C}(f_{0})]/n_{D},$$

where ${\hat{Z}}_{g}^{C}(f_{0})$ is the observed percentile of *Y_g_* in the non-cancer tissues and *I* [ ] is the indicator function equal to 1 if [ ]is true. The tail-rank statistic, *TPR_g_* ( *f*_0_), is also known as the empirical estimate of the *ROC* curve at *FPR* = *f*_0_, *ROC*( *f*_0_). Pepe et al. (2003) propose to rank genes according to *ROC* (*f*_0_). Alternatively one could fix the true positive rate at *t*_0_ say, and compare genes in regards to the corresponding false positive rates

$$FPR_{g}(t_{0})=\sum_{j=1}^{n_{c}}I[Y_{gj}^{c}\geq {\hat{Z}}_{g}^{D}(t_{0})]/n_{C}={\text{ROC}}^{-1}(t_{0}),$$

where ${\hat{Z}}_{g}^{D}$ (*t*_0_) is the (1 – *t*_0_) percentile of *Y_g_* in the cancer tissues. Lyons-Weiler et al. (2004) propose to select genes using both *TPR_g_*(*t*_0_) and *FPR_g_*(*t*_0_).

When a range of false positive (or true positive) rates is of interest, e.g. *f* ≤ *f*_0_, the corresponding true positive rates can be averaged. The average true positive rate ( *TPR**_g_*(*f*_0_)), can be written in the following equivalent ways

$$\begin{array}{l} \bar{TPR_{g}}(f_{0})=\int_{0}^{f_{0}}TPR_{g}(f)df/f_{0}\\ \;\;\;\;\;\;=\int_{0}^{f_{0}}ROC_{g}(f)df/f_{0}\\ \;\;\;\;\;\;=pAUC_{g}(f_{0})/f_{0}\\ \;\;\;\;\;\;=\text{Prob}\left[Y_{g}^{D}>Y_{g}^{C}|Y_{g}^{C}>Z_{g}^{C}(f_{0})\right] \end{array}$$

where *pAUC_g_*( *f*_0_) denotes the partial area under the *ROC* curve for gene *g* (Dodd and Pepe, 2004). This statistic is discussed in Pepe et al. (2003) for the purposes of gene selection in microarray studies. Interestingly with an unrestricted range of false positive rates, i.e. *f*_0_ = 1, the averaged true positive rate is the area under the *ROC* curve (*AUC*), also known as the expected Mann-Whitney two sample U-statistic: $AUC=\int_{0}^{1}ROC(t)dt=P(Y^{D}>Y^{C})$. Since the Mann-Whitney statistic is a simple function of the Wilcoxon rank sum statistic, it follows that using the popular nonparametric Wilcoxon statistic as the basis of gene ranking is the same as choosing the *AUC* as its basis. Lee et al. (2005) recommend using the Wilcoxon statistic to rank genes.

One important advantage of statistics like *TPR_g_*( *f*_0_), *TPR**_g_* ( *f*_0_) and *AUC* is that they are inherently com parable across genes. Clearly this is necessary for procedures that rank genes on the basis of a statistic. Their non-parametric nature implies that they do not depend on the distributions of the raw expression levels, *Y_g_*, for cancer and non-cancer tissues. They are invariant to monotone transformations of the raw data. This property makes them appealing for comparisons across genes and hence for ranking. In contrast, Welch’s *t*-statistic depends directly on the raw expression levels. For example taking the logarithm of the raw data values will change the value of *W* but it will leave *TPR_g_*( *f*_0_), *TPR*_g_ (*f*_0_) and *AUC* unchanged. Moreover, *W* weights σ*_D_* and σ*_C_* according to 1/*n_D_* and 1/*n_C_*, respectively, in its denominator. Thus, if the availability of data for two genes are such that *n_D_* or *n_C_* differ, their *W* statistics will differ even if values of *Ȳ_D_*, *Ȳ_C_*, $S_{D}^{2}$ and $S_{C}^{2}$ are the same. In our opinion it is a mistake to allow sample sizes to enter into the measure and meaning of differential expression chosen for gene ranking. The sample sizes are usually chosen by design and are affected by missingness that can vary across genes.

Interestingly, when data are normally distributed for cases and for controls, it can be shown that

$$AUC=\Phi \left[({\bar{Y}}^{D}-{\bar{Y}}^{C})/\sqrt{s_{D}^{2}+s_{C}^{2}}\right].$$

where Φ is the standard normal cumulative distribution function. Therefore, Welch’s *t*-statistic can be regarded as a scaled estimate of Φ^–1^(*AUC*) that equalizes the number of cases and controls.

In our illustration and simulations we use *TPR*(*f*_0_), and *AUC* to compare genes in regards to differential expression. In practice one may want to examine several measures of discrimination in selecting genes to study further. For example, one might seek genes for which *AUC* is large *and* for which *TPR*(0.2) is large. Although the latter statistic is not as stable as *AUC*, it is more meaningful for classification. Formal multiple hypothesis testing procedures do not consider the possibility of evaluating simultaneously multiple measures of differential expression. In the spirit of the exploratory nature of such studies however it seems that such evaluations should be encouraged.

### Evidence for differential expression

Consider the ranked list of genes, ranked on the basis of a statistic *T* (or the associated *p*-value). Randomness caused by sampling variability implies that, *T*, considered as the estimated extent of differential expression, is biased after ranking. Even if there were no differential expression for any gene, one would expect the statistics for the highest ranking genes to be large. But how large? How to calibrate the observed statistics for random chance? There are various ways one might address this. One proposal is to calculate the probability that *T*_(*g*)_ would exceed the observed value if it and all lower ranking genes were not differentially expressed. Specifically we propose to calculate the *mp*-value defined for the *g^th^* ranking gene as

$$mp-\text{value}\;(g)=P[T_{\left(g\right)}\geq t_{\left(g\right)}|H_{0j,(j)\geq g}].$$

The notation *H*_0*j*_, _(*j*)≥*g*_ means that the *mp*-value is calculated assuming that genes ranked at or below the *g^th^* are not differentially expressed. It is the probability that amongst these *m* – *g* + 1 genes, the maximum statistic, $\underset{(j)\geq g}{\text{max}(T_{j})}$ would exceed the observed value for the *g^th^* ranking gene, *t*_(*g*)_. It provides a measure of how extreme is the observed value in the setting where none of the lower ranking genes, (*j*) ≥ *g*, are differentially expressed. If the *mp*-value is low, it provides evidence that at least the *g^th^* gene must be differentially expressed.

In the classic framework of hypothesis testing, where only one statistic is under consideration (rather than many), the *p*-value has two equivalent interpretations. It is the probability that a statistic as large as that observed in the data would be observed if the null hypothesis were true. It is equivalently the lowest type I error rate for the hypothesis rejection rule that uses *T* as its basis. When multiple hypotheses and statistics are under consideration, adjusted *p*-values have been defined to generalize the latter notion. This was described in the previous section. On the other hand, our adjusted *mp*-value generalizes the former univariate concept of *p*-value to the multiple statistic setting. An appealing attribute of our *mp*-value definition is that it is not tied to any particular decision rule for rejecting hypotheses (i.e. selecting genes). It is simply a descriptive measure of evidence in favor of differential expression.

We suggest estimating *mp*-value for the *g^th^* gene using a resampling procedure that avoids assumptions about the joint distribution of test statistics and takes into account the dependence structure among genes. The resampling procedure essentially compares the observed statistic of interest with the distribution of the statistic obtained under the random condition assuming that no gene is differentially expressed. Specifically one can:

1. Compute the order statistics *t*_(1)_ ≥ *t*_(2)_ . . . ≥ *t*_(*m*)_. Let I(*g*) denote indices of genes ranking at or below the *g^th^* ranking gene.
2. Perform *B* permutations of the cancer versus non-cancer group labels and obtain {*T_j,b_*; *j* = 1, ...*m*} for each permutation sample *b*.
3. Compute the estimate

$$mp(g)=\frac{1}{B}\sum_{b=1}^{B}I\left[\underset{j\varepsilon I(g)}{\text{max}(T_{j,b})\geq t_{\left(g\right)}}\right]$$

## Data Analysis

### Simulated data

We first illustrate our methods using simulated data. The advantage of using simulated data is that we know the truth underlying the data and therefore we have a gold standard against which to compare results. Results in Table 2 are from a scenario where 100 genes are differentially expressed and 1900 genes are not. The gene expression values were simulated from a standard normal distribution for 50 controls and from either a standard normal (the 1900 non-differentially expressed genes) or a normal with mean and standard deviation equal to 1.5 (the 100 differentially expressed genes) for 50 cases. In this simulation model, expression values are statistically independent across genes.

For differentially expressed genes the true-positive rate corresponding to the positivity rule that yields a 20% false-positive rate is 67%. That is, *TPR*( *f*_0_) = 0.67 when *f*_0_ = 0.20. The area under the *ROC* curve (*AUC*) is 0.80. Table 2 shows results for subsets of the highest ranking genes, when genes were ranked according to the statistic *TPR*( *f*_0_) calculated for one simulated dataset. Interestingly, the 100 differentially expressed genes all ranked above the non-differentially expressed genes in this dataset.

First let us consider the magnitudes of the statistics. Among the top 100 ranked genes, the estimates *TPR*( *f*_0_) ranged from a minimum of 0.56 to a maximum of 0.88 and the *AUC* ranged from 0.718 to 0.912. If an investigator seeks genes for which the data suggest complete separation of cancer versus non-cancer tissues, the results are disappointing. For no gene is *TPR*( *f*_0_) = 1 or is the *AUC* = 1. Although we find the statistic *TPR*( *f*_0_) most interpretable and meaningful, gauging the potential usefulness of a *TPR*( *f*_0_) value depends entirely on the medical context under consideration. In one setting it will be vital to detect almost all cases (e.g. subjects with cancer) while in another it will be useful to detect a fraction of them. Suppose in this example that a sensitivity of at least 70% is desired when the specificity is set to 80%. There are only 49 genes for which the data suggest this level of performance. Randomness of course implies that these values are likely biased largely because they have been selected as the largest amongst a pool of 2000 genes. Nevertheless we see that it is helpful to at least view the estimated *TPR*( *f*_0_) values. The data motivate further evaluation of only the top 49 genes if the sensitivity criterion is ≥70%.

The descriptive *mp*-values that correspond to the *TPR*( *f*_0_) statistics are also displayed in Table 2. The values are very small (<0.05) for the top 90 genes, the genes with *TPR*( *f*_0_) estimates at or above 0.60. For the remaining 10 differentially expressed genes the *mp*-values are between 0.060 and 0.114. Consider the interpretation of the *mp*-value for say the 90th gene whose statistic *TPR(f*_0_*)* = 0.60. Assuming that all genes ranking at or below it are non-differentially expressed, the probability that the maximum of those *TPR* (*f*_0_) values would exceed 0.60 is only 0.03. This is strong evidence against the assumption, and in favor of differential expression at the 90th gene. Although there is strong evidence that the top 90 genes are differentially expressed, we note again that the extent of differential expression seems fairly weak for many of these genes.

The adjusted *p*-values derived from the *FDR* controlling procedures of Benjamin and Hochberg (1995) and Storey (2002) are displayed in Table 2. These also use the sensitivity statistic, *TPR*(*f*_0_), as the basis of analysis. We see that the *p*-values for the two *FDR* controlling procedures are similar, with the Storey *p*-values slightly smaller as expected. The Storey *p*-values are also smaller than our *mp*-values. It appears that rejecting the null hypothesis for all genes ranked at or higher than the 101*st* will control the expected *FDR* below 5%. In fact the *FDR* for this decision is equal to 1/101. Observe that the *FDR p*-values do not rise as sharply as the descriptive *mp*-values. Indeed, there is a remarkably sharp increase in *mp*-values at the 102*nd* ranked gene with *mp*-values >0.92 for genes ranked at or below the 103*rd*.

Ordering the genes according to the *AUC* statistic gave somewhat more powerful results. All but 2 of the 100 differently expressed genes had *mp*-values below 0.05 when analyses were based on the *AUC* statistic. The Storey *FDR* based *p*-values were below 0.05 for 104 genes, yielding an *FDR* of 4/104. For those four false-positive errors the *mp*-values were calculated as between 0.604 and 0.727 with corresponding *AUC* values between 0.678 and 0.672. These *AUC* values and their descriptive *mp*-values might be useful in deciding not to pursue further study of those genes, even though their *FDR* based *p*-values are <0.05.

In this example, we find that looking at the values of the statistical measure of differential expression and the descriptive *mp*-values leads to a more informed decision about which genes to study further than simply looking at the error controlling *p*-values alone. We also considered a slightly different simulation example (results not shown), where the differences between cases and controls were small for the expressed genes. For the 100 differentially expressed genes the distributions of cases were normal with mean 1 and standard deviation 2. In this case we find that although the Storey adjusted *p*-values deemed 79 genes as significant (<0.05), for most of them the extent of differential expression is quite weak (only 26 with *TPR* (0.20) ≥ 0.60 and for which the *mp*-values are small). This again underscores the need of taking multiple statistical components into consideration for gene selection.

### Breast cancer data

We now analyze a publicly available cDNA microarray dataset from a study of breast cancer prognosis reported by Van’t Veer et al. (2002). The data consist of approximately 25,000 gene expression measurements from 44 breast cancer patients found to have good prognosis and 34 who had a poor prognosis. The goal of the study is to identify a subset of genes that are predictive of the prognostic status of breast cancer patients. Although Van’t Veer et al. (2002) proceeded to combine data across genes for prediction, we are concerned here only with the first step to select a set of genes which are each associated with prognosis.

**Figure 1.**
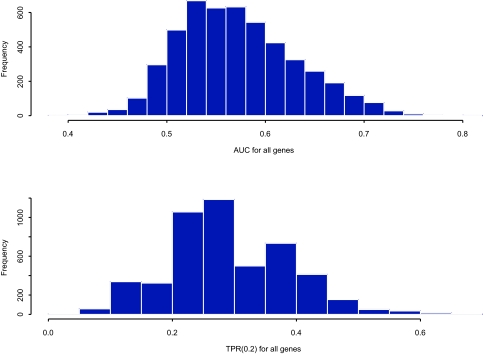
Histogram of *AUC* for all genes from the breast cancer study (top); Histogram of TPF(0.2) for all genes from the breast cancer study (bottom).

The gene expression measurement is the logarithm of the ratio of the intensities of the red to green fluorescent dyes, where green dye is used for the reference pool and red is used for the experimental tissue. In the study of Van’t Veer et al. (2002), as a first step the authors selected some 5000 genes by applying gene filtering techniques that are described in the paper. To investigate properties of our new multiple testing procedure, we follow the same gene filtering procedure and obtain a sample of 4866 genes. We use the *AUC* and *TPR*(0.20) test statistics to describe how well a gene discriminates those subjects that develop distant metastases within 5 years (poor prognosis status) from those who are disease free beyond 5 years (good prognosis status). Figure 1 displays the distribution of the *AUC*s and *TPR*(0.20) statistics for the 4866 genes.

Values ordered by *TPR*(0.20) are displayed in Table 3. When thresholds are chosen so that only 20% of controls exceed the threshold, i.e. *FPR* = 20%, the maximum *TPR* is 67.78%. Certainly none of the genes shows promise as an excellent classifier on its own. Initially it seemed surprising to us that better performance was not observed for at least one gene, even by random chance, given that almost 5,000 genes were studied. The *mp*-value of 0.009, however, indicates that even if all 5,000 genes were not differentially expressed, by random chance it would be very unlikely that the maximum *TPR*(0.20) value would exceed 67.6%.

The usefulness of the classification probabilities, *TPR*(0.20) need to be considered in light of potential clinical applications. For example, suppose the idea is to aggressively treat subjects who test positive and it is considered justifiable to treat 20% of good prognosis patients if an adequate fraction of poor prognosis patients are detected by the marker. If that ‘adequate’ fraction is 70% (i.e. *TPR*(0.20) = 0.70) then no gene appears to satisfy that criterion. If the minimally acceptable *TPR*(0.20) is 60%, then the data suggest this level of performance for 17 genes. If it is sufficient to detect 50% of poor prognosis patients, then 169 genes have estimated *TPR*(0.20) values above that level.

The descriptive *mp*-values are <0.05 for 8 genes and <0.10 for the 17 genes with *TPR*(0.20) ≥ 60%. The values do not rise so steeply as in the simulated data example, the *mp*-value being 0.346 at the gene ranked 52nd, for example. Observe that the descriptive *mp*-values are reasonably monotone in the statistic used to calculated them. This monotonicity is appealing because drawing a line in the list of genes ranked according to the descriptive *mp*-value corresponds to drawing a line on the basis of the magnitude of differential expression measure.

In contrast, the error rate controlling *p*-values are not monotone in the differential expression statistic. The reason for this is that those adjusted *p*-values are defined as functions of the raw *p*-values not directly in terms of the statistics. The raw *p*-value depends on the variability of the statistic in addition to its magnitude. For example, the variability in *TPR*(0.20) is higher for gene 196 than for gene 2348 although they both have the same estimates *TPR*(0.20) = 67.6% (see Pepe (2003) page 100 for how these variances are estimated). Thus their *p*-values differ rather dramatically. In all, 29 genes have *FDR* controlling *p*-values ≤0.05, although the corresponding genes rank from second to 183rd in terms of the statistics *TPR*(0.20) that are used to calculate the *p*-values.

Variability in the statistic is a concern. Although gene 196 appears to have the same detection rate as gene 2348, we are much less certain about it. The confidence interval would be much wider. A statistic with less variability is the *AUC*. We see that the *AUC* for gene 196 is high, (*AUC* = 0.743), that its *FDR* controlling *p*-value is very small, 0.007, and that the descriptive *p*-value is low, *mp*-value = 0.169. These facts together with its *TPR*(0.20) value and its associated *mp*-value suggest that it may be worth selecting for further study.

## Discussion

In exploratory gene expression array studies, the idea of considering multiple statistic measures for the identification of differentially expressed genes in DNA microarray have been recently explored (Hero et al. 2004; Yang et al. 2005). Similarly, we emphasize a proper analysis should evaluate multiple statistical components. The components we have mentioned are the measures of differential expression (i.e. test statistics), descriptive evidence for differential expression (i.e. *mp-*values) and the error rate controlling *p*-values. Other aspects of course will also be relevant to the rank of gene selection such as biological or epidemiological information available about the genes themselves.

The choice of statistic for quantifying differential expression is crucial, but there has been little discussion in the literature about this. We find the *TPR*( *f*_0_) particularly appealing since it is easily interpreted as the proportion of cases with values higher than the normal range of controls, a notion that is already familiar in laboratory medicine. Nevertheless other choices are valid. The important point we wish to make is that the choice deserves some thought in the analysis.

The descriptive *mp*-values that we have proposed do not necessarily give rise to procedures for designating genes as differentially expressed that have error rates controlled at specified levels. Rather, they are intuitive and descriptive and naturally generalize the univariate *p*-value concept, Prob(test statistic > observed | null), to the multivariate case. Other descriptive *p*-values could be defined. For example we also considered

$$Prob\left[T_{\left(g\right)}\geq t_{\left(g\right)}|H_{0}^{C}\right]$$

which is the probability that under the complete null hypothesis the statistic for the *g^th^* ranking gene would exceed the value observed. These probabilities tend to be very small, and can be small even if genes are not differentially expressed. Therefore we do not propose these for use in practice, but encourage the development of other measures for describing strength of evidence for differential expression.

Ideally one would summarize differential expression at a gene with a confidence interval for the magnitude of differential expression. Constructing confidence intervals is complicated by the multiplicity of genes considered simultaneously. Moreover the task of ranking genes, in effect selects genes according to the estimated magnitude of differential expression, and consequently induces bias. It is difficult to quantify the bias, particularly when genes vary in their extent of differential expression and in addition genes may be correlated.

Finally the definition of confidence interval can be generalized from the univariate definition in several ways. Paralleling definitions of *p*-values, confidence intervals that are based on controlling error rates of decision rules have been proposed (Benjamini and Yekutieli 2005). Alternative, more descriptive notions might also be considered in the future.

## Figures and Tables

**Table 1.** The classic hypothesis testing frame work for gene selection. Non-null hypotheses correspond to differentially expressed genes.

	**# not rejected**	**# rejected**	**total**
#null hypotheses	*m*_0_ – *V*_0_	*V*_0_	*m*_0_
#non-null hypotheses	*V*_1_	*S*	*m – m*_0_
total	*m–R*	*R*	*m*

**Table 2.** Simulated data for 50 cases and 50 controls. Genes are ranked using the *TPR*(0.20). Differentially expressed genes ranked 1 through 100. Non-differentially expressed genes ranked 101–2000. *P*-values based on the *TPR* statistic have superscript *T* while those based on the *AUC* statistic have superscript *A. BHp*-value uses the Benjamini-Hochberg (1995) rejection rule while *Sp*-value uses that of Storey (2002).

**Rank**	*TPR***(0.20)**	***mp*-value**^***T***^	***BHp*-value**^***T***^	***Sp*-value**^***T***^	*AUC*	***mp*-value**^***A***^	***BHp*-value**^***A***^	***Sp*-value**^***A***^
1	0.88	0	0	0	0.884	0	0	0
2	0.86	0	0	0	0.880	0	0	0
3	0.86	0	0	0	0.870	0	0	0
4	0.86	0	0	0	0.890	0	0	0
5	0.84	−0	0	0	0.864	0	0	0
6	0.84	0	0	0	0.863	0	0	0
7	0.82	0	0	0	0.869	0	0	0
8	0.8	0	0	0	0.912	0	0	0
9	0.8	0	0	0	0.886	0	0	0
10	0.8	0	0	0	0.901	0	0	0
11	0.8	0	0	0	0.858	0	0	0
12	0.8	0	0	0	0.847	0	0	0
13	0.78	0	0	0	0.869	0	0	0
88	0.6	0.03	0.003	0.003	0.752	0.006	0	0
89	0.6	0.03	0	0	0.721	0.061	0.001	0.001
90	0.6	0.03	0.004	0.004	0.771	0.003	0	0
91	0.58	0.06	0.001	0.001	0.738	0.02	0	0
92	0.58	0.06	0.004	0.004	0.752	0.006	0	0
93	0.58	0.06	0.039	0.037	0.760	0.005	0	0
94	0.56	0.113	0.025	0.024	0.678	0.604	0.021	0.02
95	0.56	0.113	0.005	0.005	0.746	0.01	0	0
96	0.56	0.114	0.018	0.017	0.751	0.006	0	0
97	0.56	0.114	0.014	0.013	0.760	0.005	0	0
98	0.56	0.114	0.005	0.004	0.734	0.023	0.001	0.001
99	0.56	0.113	0.003	0.003	0.736	0.023	0.001	0
100	0.56	0.114	0.007	0.007	0.776	0.002	0	0
101	0.52	0.375	0.010	0.010	0.645	0.985	0.119	0.116
102	0.52	0.375	0.070	0.067	0.718	0.08	0.002	0.002
103	0.46	0.924	0.157	0.150	0.615	1	0.347	0.34
104	0.46	0.925	0.083	0.080	0.653	0.96	0.08	0.079
105	0.44	0.976	0.307	0.293	0.637	0.998	0.169	0.165
106	0.44	0.976	0.791	0.755	0.614	1	0.353	0.346
107	0.44	0.976	0.124	0.118	0.573	1	0.758	0.743
108	0.44	0.976	0.402	0.383	0.576	1	0.727	0.712
109	0.44	0.976	0.321	0.306	0.617	1	0.326	0.32
110	0.42	0.997	0.619	0.591	0.608	1	0.425	0.416

**Table 3.** Results from the breast cancer prognosis study. Genes are ranked according to *TPR*(0.20) and result displayed for the top 20. The same notation as in Table 2 is used.

**Rank**	**Gene#**	**TRP(0.20)**	***mp*-value**^***T***^	***BHp*-value**^***T***^	***Sp*-value**^***T***^	*AUC*	***mp*-value**^***A***^	***BHp*-value**^***A***^	***Sp*-value**^***A***^
1	196	0.676	0.009	0.525	0.245	0.743	0.169	0.04	0.007
2	2348	0.676	0.009	0.073	0.034	0.706	0.611	0.044	0.008
3	208	0.647	0.028	0.243	0.113	0.801	0.002	0.006	0.001
4	4106	0.647	0.028	0.175	0.082	0.792	0.01	0.006	0.001
5	732	0.647	0.028	0.083	0.039	0.791	0.01	0.006	0.001
6	1823	0.647	0.028	0.011	0.005	0.744	0.164	0.04	0.007
7	4682	0.647	0.028	0.152	0.071	0.724	0.363	0.042	0.007
8	1793	0.647	0.028	0.024	0.011	0.709	0.556	0.044	0.008
9	1051	0.618	0.085	0.191	0.089	0.735	0.251	0.042	0.007
10	3816	0.618	0.085	0.595	0.277	0.725	0.353	0.042	0.007
11	3502	0.618	0.085	0.286	0.134	0.723	0.368	0.042	0.007
12	3570	0.618	0.085	0.237	0.110	0.721	0.404	0.042	0.007
13	4610	0.618	0.085	0.191	0.089	0.711	0.529	0.043	0.008
14	2332	0.618	0.085	0.011	0.005	0.700	0.684	0.048	0.009
15	1899	0.618	0.085	0.065	0.030	0.697	0.716	0.049	0.009
16	2603	0.618	0.085	0.243	0.113	0.689	0.818	0.056	0.01
17	4048	0.618	0.085	0.073	0.034	0.686	0.854	0.057	0.01
18	4698	0.588	0.172	0.008	0.004	0.762	0.062	0.032	0.006
19	917	0.588	0.172	0.49	0.228	0.739	0.21	0.04	0.007
20	936	0.588	0.172	0.274	0.128	0.732	0.274	0.042	0.007

## References

[b1-cin-03-203] Benjamini Y, Hochberg Y (1995). Controlling the False Discovery Rate: A Practical and Powerful Approach to Multiple Testing. J. Roy. Stat. Soc. Ser. B.

[b2-cin-03-203] Benjamini Y, Yekutieli D (2001). The control of the false discovery rate in multiple hypothesis testing under dependency. Ann. Statistics.

[b3-cin-03-203] Benjamini Y, Yekutieli D (2005). False Discovery Rate—Adjusted Multiple Confidence Intervals for Selected Parameters. J. Am. Statist. Assoc.

[b4-cin-03-203] Dodd L, Pepe MS (2003). Partial *AUC* estimation and regression. Biometrics.

[b5-cin-03-203] Dudoit S, Shaffer JP, Bolderick JC (2002). Multiple hypothesis testing in microarray experiments.

[b6-cin-03-203] Dudoit S, van der Laan MJ, Pollard KS (2004). Multiple testing. Part I. Single-step procedures for control of general Type I error rates. Statistical Applications in Genetics and Molecular Biology.

[b7-cin-03-203] Hero AO, Fleury G, Mears AJ, Swaroop A (2004). Multicriteria gene screening for analysis of differential expression with DNA microarrys. EURASIP Journal on Applied Signal Processing,.

[b8-cin-03-203] Lee JW, Lee JB, Park M, Song SH (2005). An extensive comparison of recent classification tests applied to microarray data. Comput. Stat Data. Analysis.

[b9-cin-03-203] Lehmann EL, Romano J (2004). Testing Statistical Hypotheses.

[b10-cin-03-203] Lyons-Weiler J, Patel S, Becich MJ, Godfrey TE (2004). Tests for finding complex patterns of differential expression in cancers: towards individualized medicine. BMC Bioinformatics.

[b11-cin-03-203] Pepe MS (2003). The Statistical Evaluation of Medical Tests for Classification and Prediction.

[b12-cin-03-203] Pepe MS, Longton G, Anderson G, Schummer M (2003). Selecting differentially expressed genes from microarray experiments. Biometrics.

[b13-cin-03-203] Storey JD (2002). A direct approach to false discovery rates. J. Roy. Stat. Soc. Ser. B.

[b14-cin-03-203] van der Laan MJ, Dudoit S, Pollard KS (2004). Augmentation procedures for control of the generalized family-wise error rate and tail probabilities for the proportion of false positives. Statistical Applications in Genetics and Molecular Biology.

[b15-cin-03-203] van der Laan MJ, Dudoit S, Pollard KS (2004). Multiple testing. Part II. Step-down procedures for control of the family-wise error rate. Statistical Applications in Genetics and Molecular Biology.

[b16-cin-03-203] van’t Veer LJ, Dai H, van de Vijver MJ, He YD, Hart AAM (2002). Gene expression profiling predicts clinical outcome of breast cancer. Nature.

[b17-cin-03-203] Westfall PH, Young SS (1993). Resampling-based multiple testing: examples and methods for p-value adjustment.

[b18-cin-03-203] Yang YH, Xiao Y, Segal M (2005). Identifying differentially expressed genes from microarray experiments via statistic synthesis. Bioinformatics.

